# Integrating numerical modelling and scenario-based sensitivity analysis for saltwater intrusion management: case study of a complex heterogeneous island aquifer system

**DOI:** 10.1007/s10661-023-11159-z

**Published:** 2023-04-11

**Authors:** Ashneel Sharan, Bithin Datta, Alvin Lal

**Affiliations:** 1 Discipline of Civil Engineering, College of Science & Engineering, James Cook University, Townsville, Queensland 4811 Australia; 2 Global Centre for Environmental Remediation, College of Engineering, Science and Environment, The University of Newcastle, Callaghan, NSW 2308 Australia

**Keywords:** SEAWAT, Heterogeneous, Complex aquifer, Pacific island countries, Sensitivity analysis, Saltwater intrusion

## Abstract

Population growth, industrialisation and increasing agricultural demands have significantly stressed groundwater resources in Pacific Island countries (PICs). Climate change and sea-level rise also affect the groundwater resources in PICs. These anthropogenic and natural factors give rise to saltwater intrusion (SWI), a major growing environmental problem in the PICs. SWI is a highly non-linear process which makes it more complex to manage. However, with the help of numerical modelling, SWI can be monitored, managed and controlled. In the present study, we used an illustrative study area where the hydrogeological parameters and other boundary conditions used are similar to the PICs aquifer systems in Vanuatu. The scenarios include changing the barrier wells, injection wells, recharge, hydraulic head, hydraulic conductivity and grid size. The numerical simulation model of the study area was developed, and different scenarios were tested using SEAWAT modules. Apart from salt, we also modelled leachate and engine oil present in the investigated study area to see how it affects the freshwater wells over time. The scenario-based sensitivity analysis tests indicate that injection wells, recharge and hydraulic conductivities are highly sensitive, and with the proper modification, SWI can be managed or regulated. The sensitivity of grid size showed that the simulated results varied within the 10% range of different gird sizes. Moreover, it was also found that the rise in sea level or coastal heads by 0.3–1 m does not significantly cause further SWI encroachment in aquifers. The results from this study are very crucial in this modern era when freshwater needs in coastal areas, especially PICs, are rapidly increasing, and fresh groundwater resources are declining. The novel outcome presented in this study opens pathways for further detailed modelling and numerical studies in the field of SWI management strategy development and is, therefore, beneficial for policymakers, groundwater modellers and general scientific communities.

## Introduction

Groundwater is the oldest and most widely used water resource on Earth, whereby 35% of water is used by humans supplied through sub-surface aquifers worldwide (Frankel, 2015). Groundwater usage has increased in the last few decades merely due to agricultural demands, population growth and industrialisation (Hamed et al., 2018; Howard, 2015). The increase in demand for groundwater resources has imposed a lot of stress and environmental problems in coastal aquifers across the globe. Saltwater intrusion (SWI) is one of the major environmental problems faced by coastal aquifers due to the over-abstraction of groundwater (Alley et al., 1999; Michael et al., 2017; Sharan et al., 2021). SWI occurs when the density of the groundwater is less than that of seawater when they hydraulically interact, forming depression and ascension zones. SWI is also described as the degradation or contamination of groundwater when the salinity of freshwater aquifers increases. Freshwater is identified by determining the total dissolved solids (TDS) present in the water. Freshwater has TDS < 1000 mg/L, whereas salty water has TDS > 35,000 mg/L. Table 1 shows the general salinity classifications of different water types.

**Table 1:** General salinity classification (Hillel, 2000)

Water type	Total dissolved solids (mg/L)	Electrical conductivity (μS/cm)	General usage
Rainfall	≤ 20	≤ 30	Drinking and all irrigation, also suitable for all livestock
Wastewater effluents	250–800	373–1194	Drinking and all irrigation, also suitable for all livestock
Freshwater	< 1000	< 1500	Drinking and all irrigation, also suitable for all livestock
Brackish water	1000–10,000	1501–15,000	Not suitable for drinking, but used for aquaculture, cooling water for power generation Saline water (TDS = 2000–10,000 mg/L)
Seawater	> 35,000	> 52,000	Not suitable for drinking or irrigation, also known as brine water, can be used for limited mining or industrial purposes

Pacific Island countries (PICs) use groundwater daily for drinking, cooking, washing, farming, irrigation, etc. Climate change, natural disasters, over-abstraction and other anthropogenic factors constantly affect the groundwater resources in PICs (Sharan et al., 2021). The groundwater resources in PICs are also prone to SWI problems. Hence, there is a need for groundwater modelling tools to tackle the SWI in the coastal aquifers in PICs. Table 2 summarises the twelve PICs profile, their water resources and the significant contaminants affecting their water resources. The summary vividly indicates that SWI is a major issue in small island nations affecting their coastal aquifers. The studies conducted in managing the SWI problems are very limited in all the PICs except for Kiribati, where the research is active with some published reports and papers.

**Table 2:** Pacific Island countries profile and water resources

Country^a^	Approximate population (in 2020)^a^	Land area (km sq.)^a^	Island geology^b^	Major source of income^c^	Major water sources^c^	Major water contaminants or problems^c^	Saltwater intrusion studies^c^
Cook Islands	17,595	236	Atoll, volcanic, volcanic and limestone	Tourism, agriculture, pearls	Desalination, groundwater, rain and surface water	Septic tank leakage, animal and human waste SWI	Very limited
Fiji	909,466	18,272	Volcanic, limestone, atoll	Tourism, sugarcane, agriculture	Groundwater and surface water	Over-abstraction of groundwater, waste disposals, SWI	Very limited
Kiribati	123,419	811	Majority atoll, 1 limestone	Foreign aid, seafood exports, copra	Desalination, groundwater, rainwater	SWI, animal waste, droughts, over-abstraction., SWI	Active
Nauru	10,903	21	Limestone	Foreign aid, seafood export, tourism, agriculture	Desalination, groundwater, rainwater	Sewage, long droughts, over-abstraction, SWI	Very limited
Niue	1648	260	Limestone	Agriculture	Groundwater	Animal and human waste, over-abstraction	Very limited
Palau	18,233	459	Limestone, volcanic	Agriculture and Seafood	Groundwater, rain and surface water	Deforestation, sediment erosion, pesticides, human and animal waste	Very limited
Papua New Guinea (PNG)	9,292,169	46,2840	Atolls, coral Is., volcanic, limestone	Agriculture, mining, crude oil	Groundwater, rain and surface water	Farm chemicals, fertilizers, over-abstraction, SWI	Very limited
Samoa	202,239	2842	Volcanic	Agriculture, seafood export, tourism, copra	Groundwater and surface water	sediment erosion, human and animal waste, SWI	Very limited
Solomon Islands	721,159	28,896	Atolls, volcanic, limestone	Log exports	Groundwater, rainwater	Animal and human waste, over-abstraction, SWI, logging	Very limited
Tonga	107,749	747	Volcanic, limestone, limestone and sand, mixed	Agriculture, tourism	Groundwater	Farm chemicals, animal waste, fertilizers, over-abstraction, SWI	Very limited
Tuvalu	12,066	26	Atoll	Fishing licence to foreign vessels	Groundwater and rainwater	Highly contaminated groundwater, animal, factory, human waste, chemicals, erosion, SWI	Very limited
Vanuatu	321,832	12,189	Majority volcanic, sand and limestone	Agriculture, seafood export, tourism	Groundwater and rainwater	SWI, animal, factory, human waste, chemicals	Very limited

Source: ^a^World Population Review (2022)

^b^Falkland (2002)

^c^Sharan et al. (2021)

Coastal aquifers need to be monitored frequently, and it is essential to know the exact landward extent of saline water and freshwater discharge to maintain the saltwater-freshwater interface at equilibrium. There are numerous techniques and tools for preventing, managing and minimising the SWIs. The detailed techniques and tools are thoroughly described by Sharan et al. (2021). Three-dimensional (3D) numerical modelling techniques are one of the reliable, efficient and cost-effective tools for preventing, managing and minimising the SWIs in coastal aquifers (Lal & Datta, 2017; Roy & Datta, 2020; Sharan et al., 2021, 2023). PICs have used a limited number of numerical models to manage the SWI. Some of the 3D numerical models used in PICs groundwater management include SEAWAT (Bosserelle et al., 2015), SUTRA (Kumar, 2016) and FEMWATER (Lal & Datta, 2018, 2019). A SEAWAT-based numerical model has only been developed for the Bonriki aquifer (1.385° N, 173.144° E) in Kiribati to manage SWIs. However, more work still needs to be done, and the complexity of the conceptual model needs to be considered for the Bonriki aquifer. The SWI is a non-linear process. Hence, developing a complex model and using all possible boundary conditions will give optimum SWI management strategies.

SEAWAT-based numerical simulation models are developed using finite difference techniques. SEAWAT code is used for complex geometrics, and geological settings, including SWI, submarine groundwater discharge, brine transport and groundwater flow near salt domes (Cobaner et al., 2012). Guo et al. (2019) studied the effects of sea-level fluctuations, groundwater pumping, and inland recharge on SWI in complex coastal aquifers. The authors developed the numerical model using SEAWAT and calibrated the hydrogeological parameters using real-time data. The authors found that their simulated groundwater level and salt concentrations were comparable with the results from the observation wells. Moreover, they also reported that high and low tide portrays a major role in SWI in coastal aquifers.

Hagagg (2019) used the SEAWAT code to examine the level of SWI in the Karstic aquifer of El Negila (30.75° N, 30.698° E). The author tested the scenarios, including increased sea-level heights and pumping activities for 2022. The author reported that the SWI would increase if the pumping rates and sea level heights increased. Hamidi et al. (2021) used the SEAWAT code to model the SWI for the Rmel-Oulad Ogbane coastal aquifer in Larache, Morocco. The authors focused on two scenarios which include climate change and sea-level rise (SLR). The authors reported that the over-pumping of groundwater and SRL caused by climate change are the primary drivers of SWI in coastal aquifers. Moreover, the authors also reported that climate change also reduces the natural recharge, which eventually reduces the fresh groundwater resources.

The SLR, climate change and over-abstraction of groundwater affect the quantity and quality of groundwater resources in PICs. These factors also cause SWI in coastal aquifers. Hence, managing the anthropogenic and natural factors affecting groundwater resources is very important. A numerical modelling approach is considered for this study to establish proper links between climate change, SLR and over-abstraction. This study will test different scenarios, and sensitivity analysis will be conducted by changing various parameters in our model domain. Moreover, multi-species contaminants will be introduced and modelled within our model domain. To the best of our knowledge, some combinations of different scenarios tested in this study have never been investigated in previous studies. Moreover, the combined SWI and other species flow and transport will be the first for PICs. This study will be beneficial to hydrogeologists and groundwater professionals in considering various scenarios while developing their models using SEAWAT. Moreover, this study will provide better scenarios capable of managing SWIs. Also, this study will show how multi-species contaminants are transported in coastal aquifers using SEAWAT, which will benefit lawmakers in drawing up policies for controlling these contaminant species before it contaminates the freshwater wells.

## Methods

Saltwater intrusion is a complex non-linear process that requires mathematical models like numerical models to build the case simulation and provide management strategies for controlling saltwater intrusion. The numerical simulation model was developed using SEAWAT modules in groundwater modelling system (GMS). SEAWAT numerical code was developed to simulate 3D, variable density, transient groundwater flow in porous medium combining MODFLOW and MT3DMS programs. MODFLOW solves the non-linear variable density flow equations, and MT3DMS solves the non-linear temporal and spatial salt or solute transport equations (Guo & Langevin, 2002).

## Governing equations

The variable-density flow process solves the following form of the variable-density groundwater flow equation (tensors and vectors are shone in bold) (Langevin et al., 2008):

1 $$\begin{aligned}&\nabla \bullet \left[\rho \frac{{\mu }_{0}}{\mu }{{\varvec{K}}}_{0}\left( \nabla {h}_{0}+ \frac{\rho - {\rho }_{0}}{{\rho }_{0}} \nabla z\right)\right]\\&\quad = \rho {S}_{s,0} \frac{\partial {h}_{0}}{\partial t}+ \theta \frac{\partial \rho }{\partial C} \frac{\partial C}{\partial t}- {\rho }_{s}{q}_{s}^{^{\prime}}\end{aligned}$$

where ${\rho }_{0}$ is the fluid density (kg/m^3^) at the reference concentration and reference temperature; *μ* is dynamic viscosity (kg/md); ***K***_**0**_ is the hydraulic conductivity tensor of material saturated with the reference fluid (m/day); *h*_0_ is the hydraulic head (m) measured in terms of the reference fluid of a specified concentration and temperature (as the reference fluid is commonly freshwater); *S*_*s,*0_ is the specific storage (1/m), defined as the volume of water released from storage per unit volume per unit decline of *h*_*0*_; *t* is time (day); $\theta$ is porosity (dimensionless); *C* is salt concentration (kg/m^3^); and *q'*_*s*_ is a source or sink (1/day) of fluid with density ${\rho }_{s}$.

The integrated MT3DMS Transport process solves the following form of the solute transport equation:

2 $$\begin{aligned}\left(1+ \frac{{\rho }_{b}{K}_{d}^{k}}{\theta } \right)\frac{\partial ( \theta {C}^{k} )}{\partial t}=&\ \nabla \bullet \left(\theta {\varvec{D}}\bullet \nabla {C}^{k} \right)- \nabla \bullet \left({\varvec{q}}{C}^{k}\right)\\&- {q}_{s}^{^{\prime}} {C}_{s}^{k}+\sum_{k=1}^{N}{R}_{k}\end{aligned},$$

where ${\rho }_{b}$ is the bulk density (mass of the solids divided by the total volume) (mg/m^3^), ${K}_{d}^{k}$ is the distribution coefficient of species *k* (m^3^/kg), ${C}^{k}$ is the concentration of species *k* (kg/m), ***D*** is the hydrodynamic dispersion coefficient tensor (m^2^/d), ***q*** is specific discharge (m/day), and ${C}_{s}^{k}$ is the source or sink concentration (kg/m^3^) of species *k*. ${R}_{k}$ (*k* = 1, … *N*) is the chemical reaction term (kg/m^3^day) of species *k*.

In previous versions of SEAWAT, $\frac{{\mu }_{0}}{\mu }$ was approximated as one, and thus, viscosity effects were neglected, and fluid density was treated as a simple linear function of only one solute species. In SEAWAT version 4, fluid density and viscosity can be calculated using concentrations from one or more solute species. These equations of state relate the density and viscosity terms in Eq. 1 to one or more of the MT3DMS species concentrations (*C *^*k*^) in Eq. 2. By allowing one or more of the MT3DMS species to affect fluid density and viscosity, variable-density groundwater flow can be coupled with simultaneous solute and heat transport (Langevin et al., 2008). Moreover, the previous version of SEAWAT and variable density groundwater flow and transport equations are given in by Guo and Langevin (2002) and Zheng and Wang (1999).

## Study area

**Fig. 1:**
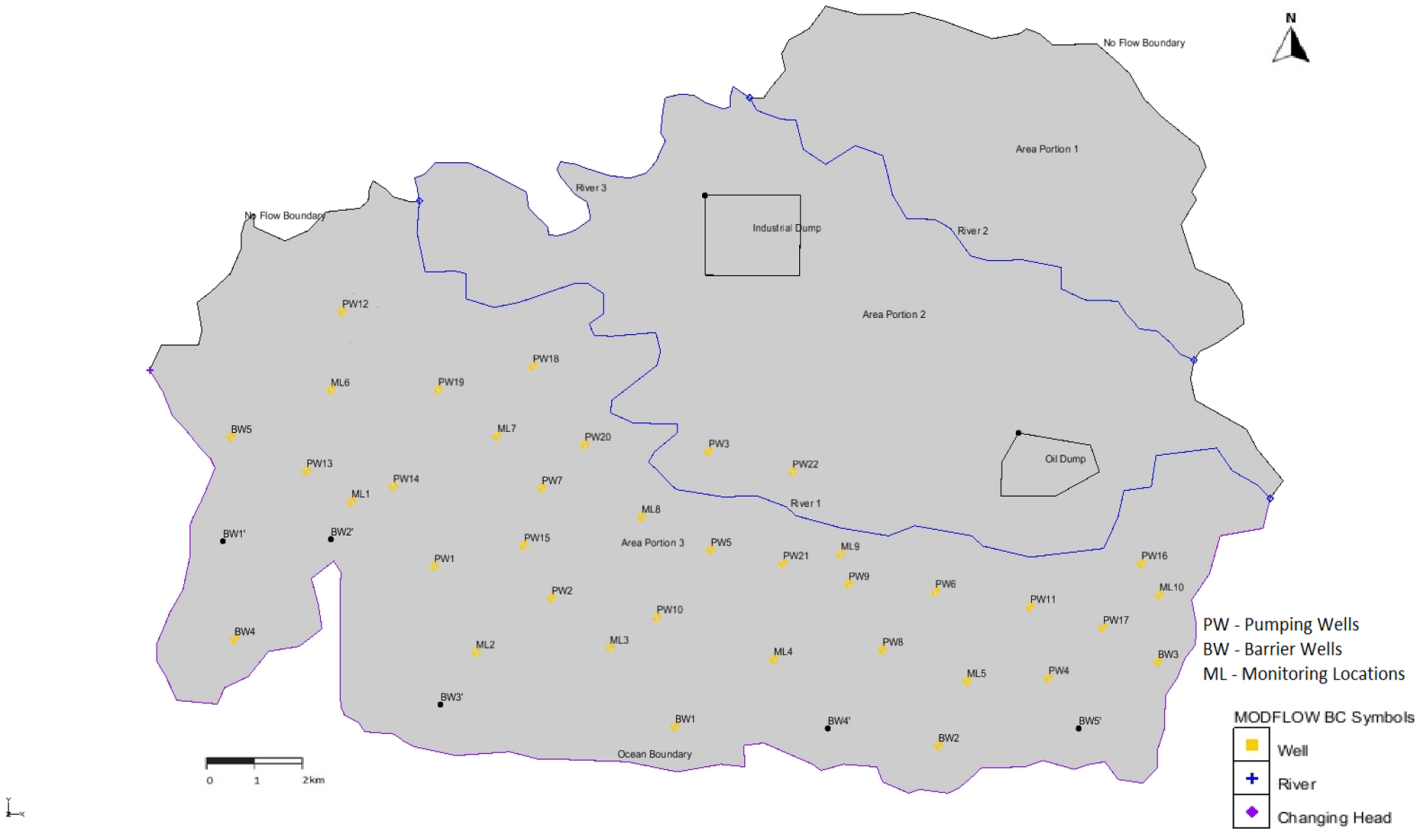
Plan view of the study area

An illustrative study area representing an island aquifer system was used to simulate the 3D saltwater intrusion process and test different SWI management scenarios. The one-layer unconfined aquifer used in this study was 125.58 km^2^. The aquifer system considered was complex, heterogeneous and isotropic in nature, with different values of hydraulic conductivities and porosities in the horizontal direction. The hydraulic conductivities for area portions 1, 2 and 3 are 10 m/day, 15 m/day and 20 m/day, respectively. Also, the porosities for area portions 1, 2 and 3 are 0.2, 0.28 and 0.32, respectively. The study area consisted of an ocean boundary, three rivers, pumping wells, barrier wells, monitoring wells, industrial waste and oil dumping sites, as shown in Fig. 1. The dumping site is assumed to be leaking at a prolonged rate, whereby the leachate and oil are seeping into the groundwater system. The annual rainfall for the area was 115 in/year. The illustrative study area is an actual groundwater pumping site in Vanuatu (15.3767° S, 166.9592° E) in Pacific Island countries (PICs). PICs do not have enough groundwater data to calibrate and validate groundwater models. Due to the unavailability of the hydraulic heads, groundwater pumping and water concentrations data, we treated the area as an illustrative study area utilising the field data to the extent available to resemble the PICs aquifer system. The dumping site and the number of wells and rivers created in the model domain are added to introduce more complexity to the model. However, some boundary conditions and hydrogeological parameters are realistic and taken from the actual study area in Vanuatu.

### Model discretisation

The MODFLOW-2000 module was used to develop the conceptual model domain of the current study area. The length of the study area in the *X*-direction was 13,822.15 m, the length in *Y*-direction was 9083.80 m, and the length in *Z*-direction was 100 m. The cell size used to create the finite-difference grids was 50 m × 50 m. The number of nodes was 1,010,748, and the number of cells was 50,414. The grid top elevation was 200 m, and the grid bottom elevation was 100 m.

### Numerical model and boundary conditions

The study area was divided into three small area portions representing an island aquifer system. Area portion 1 had fine to medium-grained sand, area portion 2 had coarse-grained sand, and area portion 3 had sand/gravelly sand. The top boundary was a free surface, and the bottom boundary was treated as a no-flow boundary. There were three no-flow boundaries within the model domain. One was between rivers 1 and 2, the other was towards the north-eastern side, and the third no-flow boundary was on the western side of the model domain. The boundary on the south side of the model domain was the coastal side boundary, and it was considered the specified head (constant head boundary), with the heads being at 181 m each for two different nodes. River 1 head value of the eastern boundary was 182 m, and 190 m of the northwestern boundary. River 2 head value of the eastern boundary was 188 m, and 195 m of the northwestern boundary. River 3 head value on the western side was 190 m, whereas 195 m was on the north-eastern boundary. The thickness of the aquifer was 95 m. Twenty-two pumping wells and 10 monitoring location wells were uniformly placed along the model domain. The barrier wells and injection wells were also introduced in the model domain after testing different SWI management strategies. Pumping wells act as the sink to the system, whereas injection wells can be considered as sources to the system. The uniform pumping of 10,000 m^3^/day from 22 pumping wells was considered. The pumping rates and locations of production wells were not changed because we wanted to compare the best scenarios for managing the saltwater intrusions. Also, changing the pumping rates could have increased our simulations, and our numerical modelling would have become computationally expensive. However, the rates of pumping have been altered by adding barrier wells and injection wells. Recharge was also used as a source to the model domain uniformly. The recharge rates and other hydrogeological parameters used in the development of the model are given in Table 3.

**Table 3:** Model parameters and their values used in the numerical simulations

Definition	Values	S.I. Units
Area portion 1 (fine to medium-grained sand)	Hydraulic conductivity, *K* = 10 Porosity = 0.2	*K* = m/day Porosity = dimensionless
Area portion 2 (coarse-grained sand)	Hydraulic conductivity, *K* = 15 Porosity = 0.28	
Area portion 3 (sand/gravelly sand)	Hydraulic conductivity, *K* = 20 Porosity = 0.32	
Horizontal anisotropy	1	Dimensionless
Freshwater density	1000	kg/m^3^
Seawater density	1025	kg/m^3^
Saltwater concentration	35	kg/m^3^
Specific storage	0.0035	1/m
River conductance	1	m^2^/dm
Longitudinal dispersivity	20	m
Recharge	0.0004	m/day
Stress period	30	years
Density/con. constant, F	0.7143	dimensionless
Leachate recharge concentration	30,000	kg/m^3^
Bulk density	60,000	kg/m^3^
Oil recharge concentration	20,000	kg/m^3^
Leachate dissolved rate	0.00005	1/day
Leachate sorbed rate	0.00005	1/day
Leachate sorption const	0.000006	m^3^/kg
Oil sorption const	0.000003	m^3^/kg
Oil dissolved rate	0.00008	1/day
Oil sorbed Rate	0.00008	1/day

The conceptual model was developed and simulated in a steady-state using MODFLOW. The simulated heads were then used as initial heads for the transient simulation in MT3DMS and SEAWAT. The maximum stress period used was 10,950 days. We simulated the hydraulic head values in a steady state, until the difference in heads was not large. Then, we used these as initial heads in transient simulations. The initial salt concentrations were assumed to be zero as we did not have any salinity data from Vanuatu. If we had some data, then we could have used that to simulate our initial concentrations. The advection, dispersion, source and sink mixing packages and chemical reaction packages were used for solute transport. The species that are simulated are defined as salt in the MODFLOW and MT3DMS modules. Once the flow and solute modules were ready, we initiated SEAWAT-version 4 modules using the variable-density flow (VDF) package. To calculate the internodal density within the model domain, we used an upstream-weighted algorithm within the SEAWAT module. The freshwater density was used as the reference fluid density in the SEAWAT module. The density and concentration slope constant factor *F* was also used to prevent the model to over-estimate the freshwater lens. If we treat *F* = 0, then as the concentration changes, there will be no change in the density. Hence, the results will be similar to the results simulated by MT3DMS. Hence, SEAWAT was used to generate the results because it uses VDF (freshwater and seawater) to make the freshwater lens difficult to circulate freely, forming a zone of transition.

### Different scenarios tested

The numerical simulation model was developed using the SEAWAT module, and the different scenarios were tested to find the best SWI management strategies in coastal aquifers. Seven different scenarios were tested, and the sensitivity analysis was done to see how the model would behave if different parameters were changed. The different scenarios include the changes in barrier wells, injection wells, hydraulic conductivities and porosities, recharge rates, hydraulic heads, multi-species and grid size. The finite-difference method was to discretise the grids. The finite-difference grid method allows us to change different parameters without changing the grid size and node locations. However, if we use the finite-element method, it is challenging to test different scenarios using different parameters because every time, the mesh will change, eventually changing the mesh size and node locations. Hence, conducting the sensitivity analysis becomes more challenging.

## Results

### Scenario 1: Increasing the number of barrier wells

The current study area had 5 barrier wells (BWs) placed uniformly near the ocean boundary. In this scenario, the number of BWs was increased by 50%. The 5 additional BWs were also placed uniformly in the model domain along the ocean boundary and marked as BW′, as shown in Fig. 1. The study area was initially modelled without using any BWs. It was also modelled using 5 BWs and then increasing the number of BWs by 50%. The results did not show significant variations in the salt concentrations in either of the situations. Hence, we simulated our model using 5 BWs and tested how the salt concentration at various MLs would change if we increased the BWs at uniform locations. The model domain was mapped with 10 BWs, and all the other boundary conditions and hydrogeological parameters remained the same. The BWs acted as the sink to the system, and the pumping rate of the 5 BWs was considered as 12,000 m^3^/day.

SEAWAT simulation was carried out, and the result was recorded at various time steps. The plan view of the 3-time steps (10, 20, 30 years) with the 50% increased BWs is shown in Fig. 2(b-10 to b-30). Figure 2 (a-10 to a-30) show the base case results or the controlled simulated results. (Figure 3)The movement of saline water towards the PWs is clearly shown in the different time steps. The side view at ML1 was also recorded, and the results are shown in Fig. 4(a-10–a-30). The graph clearly shows how the SWI occurs at ML1 in 30 years. The variations in the salt concentration in the model domain are shown in the plan view and the side view, Figs. 2(b-10 to b-30) and 4(b-10 to b-30), respectively. The figures with the numbers after the letter indicate the year to which the simulated result corresponds. The side view of this scenario was taken at ML 1. Hence, Figs. 5a and 4(b-10 to b-30) show how the concentration was decreased at ML 1. However, seaward of ML 1, the concentration was ≈ 39.5 kg/m^3^, shown in red.

**Fig. 2:**
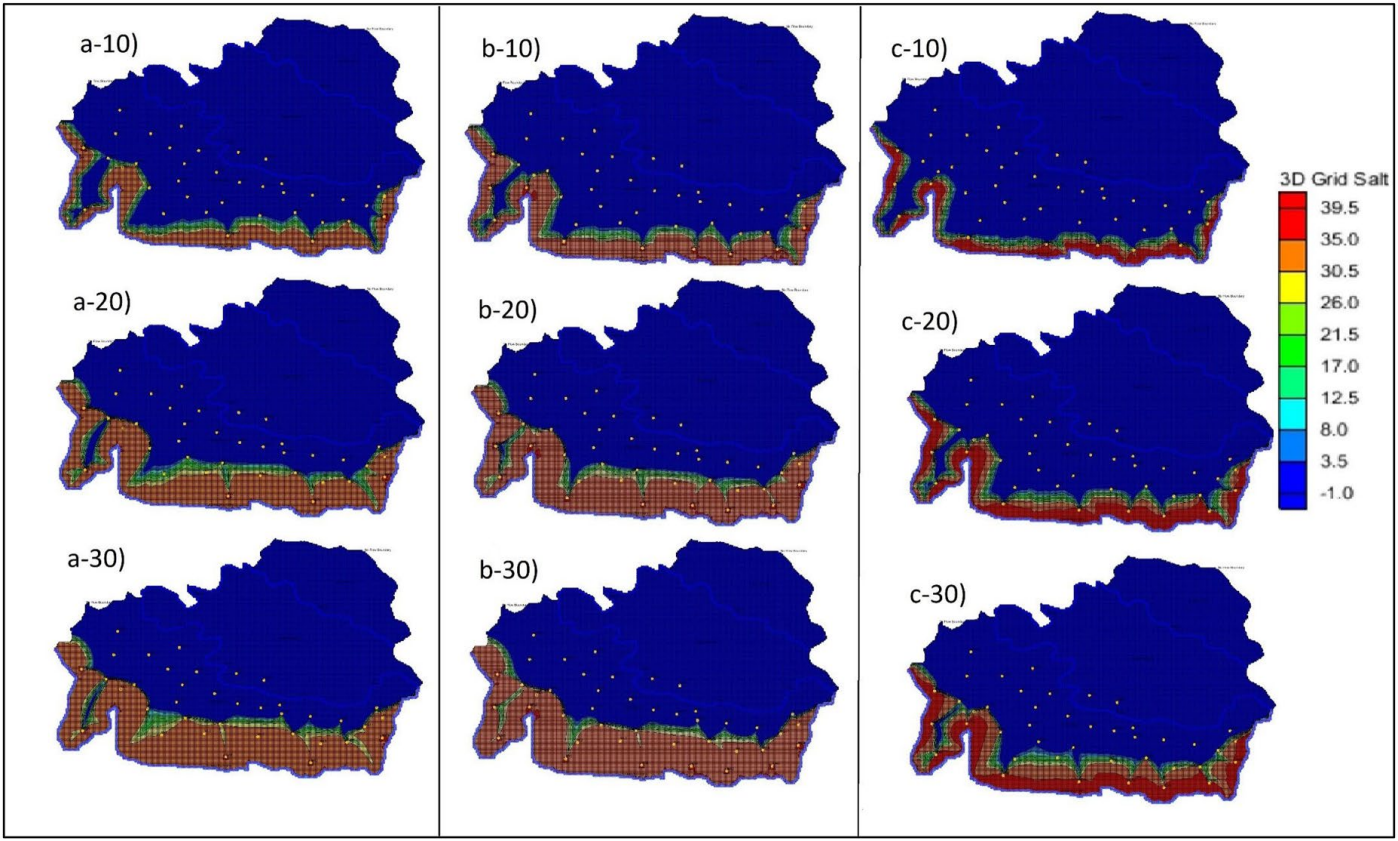
(**a-10**–**a-30**), (**b-10**–**b-30**) and (**c-10**–**c-30**) show the plan view of the simulated results of saltwater intrusion for the controlled area, the addition of barrier wells (scenario 1) and increasing the hydraulic conductivities and porosities (scenario 2), respectively. The -10, -20 and -30 represent the time steps 10, 20 and 30 years, respectively

**Fig. 3:**
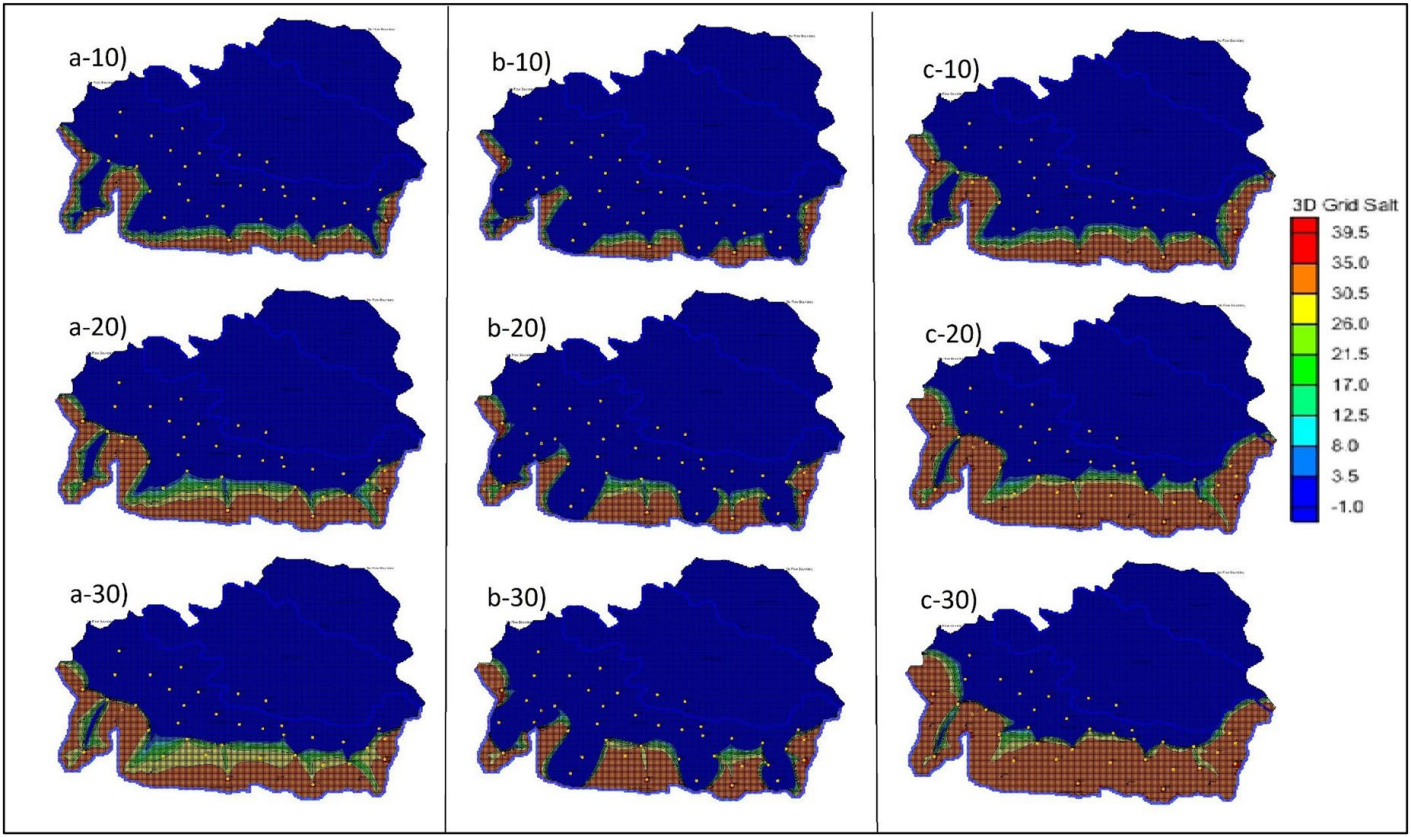
(**a-10**–**a-30**), (**b-10**–**b-30**) and (**c-10**–**c-30**) show the plan view of the simulated results of saltwater intrusion for changing recharge (scenario 3), the addition of injection wells (scenario 4) and increasing the hydraulic heads (scenario 5), respectively. The -10, -20 and -30 represent the time steps 10, 20 and 30 years, respectively

**Fig. 4:**
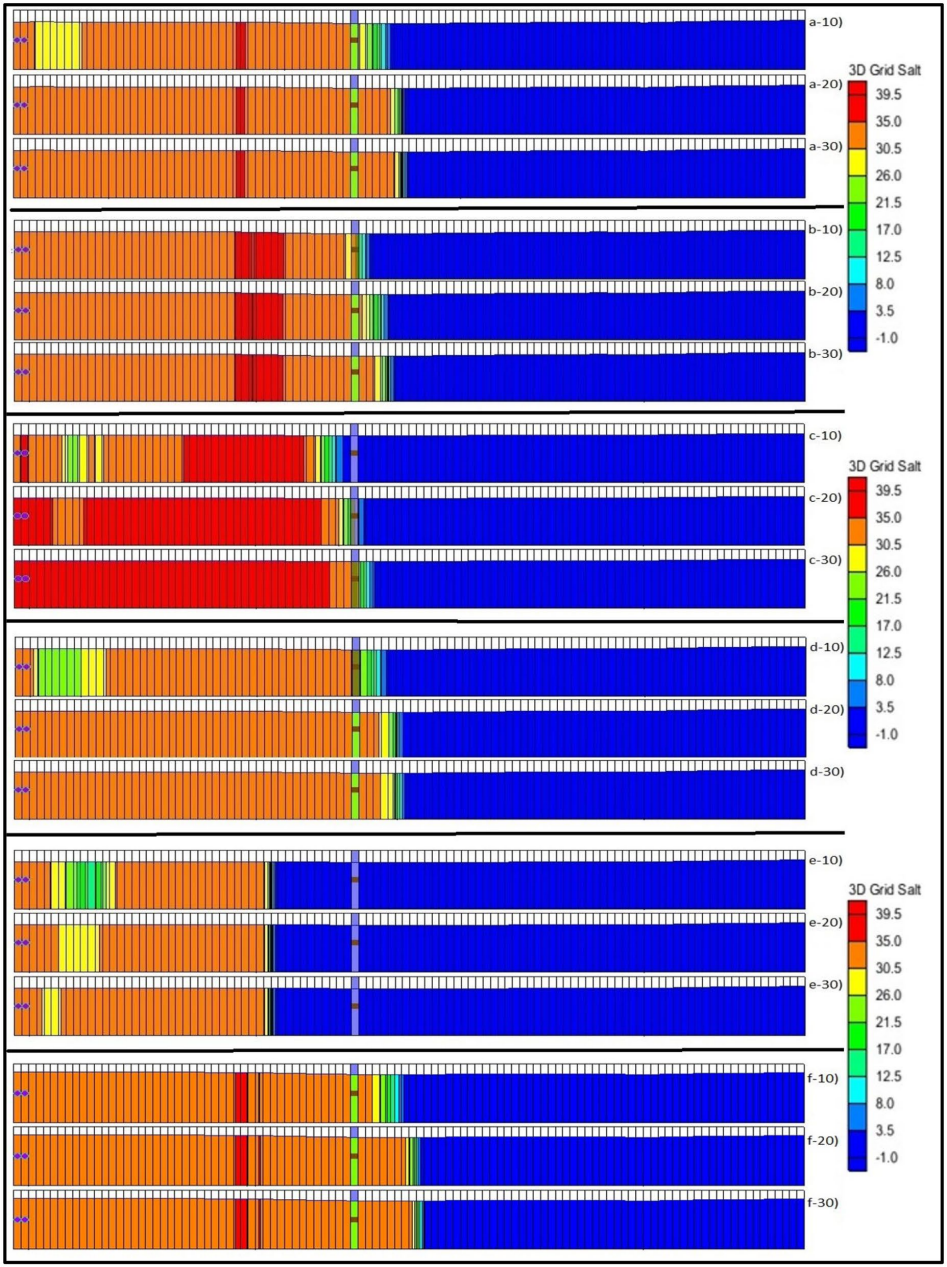
(a-10–a-30), (b-10–b-30), (c-10–c-30), (d-10–d-30), (e-10–e-30) and (f-10–f-30) show the side view of the simulated results of saltwater intrusion at monitoring location 1 for the controlled area, the addition of barrier wells (scenario 1) and increasing the hydraulic conductivities and porosities (scenario 2), changing recharge (scenario 3), the addition of injection wells (scenario 4) and increasing the hydraulic heads (scenario 5), respectively. The -10, -20 and -30 represent the time steps 10, 20 and 30 years, respectively

**Fig. 5:**
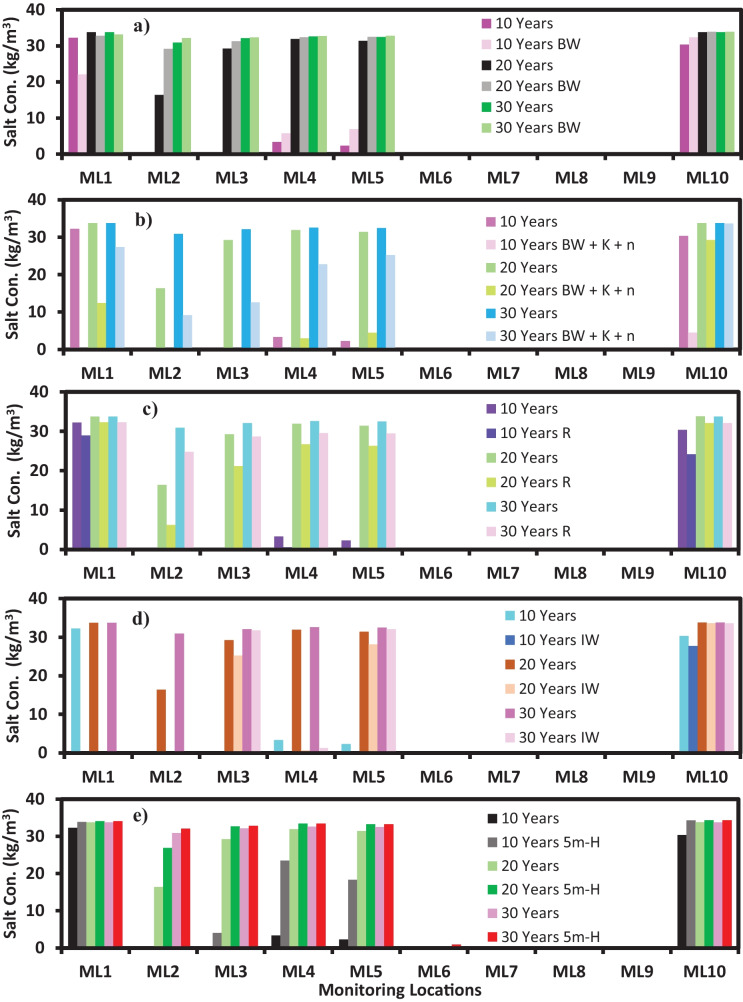
**a**–**e** show the comparison of simulated salt concentrations at various monitoring locations for the controlled area against the addition of barrier wells (scenario 1), increasing the hydraulic conductivities and porosities (scenario 2), changing recharge (scenario 3), the addition of injection wells (scenario 4) and increasing the hydraulic heads (scenario 5), respectively

Moreover, 10 monitoring location wells (ML) were uniformly placed in the model domain to record the salt concentration values. The salt concentration after 10 years, 20 years and 30 years was plotted against the salt concentrations of these MLs without BW′ and any changes in the boundary conditions or hydrogeological parameters. To compare the changes in the salt concentrations at various MLs, a bar graph was plotted using the concentrations at 10 MLs with the new simulated values and the initial simulated values referred to as controlled values for this research. The comparison of different salt concentrations at different MLs is shown in Fig. 5a. MLs 1–5 and 10 showed the changes in the concentrations, whereas MLs 6–9 did not show significant changes bearing the concentration values close to 0 kg/m^3^.

The salt concentrations for ML 1 at 10–20-30-year time step were decreased by 31.57%, 2.85% and 1.76%, respectively. For ML 2, the salt concentration at the 10-year time step was 0.054 kg/m^3^, whereas the salt concentration for the initial set-up was 0.00053 kg/m^3^. The 20-year time step and 30-year time step show 78.05% and 4.03% increase in salinity, respectively. For ML 3, no significant changes were noted for the 10-year time step, whereas 20-year and 30-year time steps show a 7.11% and 0.77% increase in salinity. For ML 4, 72.58%, 1.53% and 0.90% increments of salt concentration were noted for 10–20-30-year time steps, respectively. Similarly, for ML 5 and 10, the 10-year time step showed a significant increment of salt concentration, whereas the increase was relatively small for 20- and 30-year time steps.

### Scenario 2: Increasing the hydraulic conductivity, porosity and BWs

The hydraulic conductivities and porosities in different area portions were increased by 50% from the initial values. The new hydraulic conductivities for area portions 1 to 3 were 20 m/day, 30 m/day and 40 m/day, respectively. The new porosity values for area portions 1 to 3 were 0.4, 0.54 and 0.64, respectively. Also, the extra BWs which were introduced in scenario 1 were also maintained in scenario 2. The plan view of the salt concentrations at different time steps for scenario 2 is shown in Fig. 2(c-10 to c-30). From the graph, we can see that the saltwater flow into the aquifer from 10 to 30 years is reduced. However, the salt concentrations near the coastline have increased to a value of 39 kg/m^3^. In addition, the saltwater did not reach PWs 1, 2, 8 and 10. Whereas, in the scenarios without changing the hydraulic conductivity and porosity, it shows the saltwater encroachments of the PWs close to the coastline.

The side view of scenario 2 was viewed from ML 1, and the results are shown in Fig. 4(c-10 to c-30). From the graph, it can be seen that as the hydraulic conductivity and porosity increase, the saltwater concentration decreases. Also, the salt concentration towards the southwest of ML 1 has increased, as shown in dark red colours. These salt concentrations and SWI can be compared with the side view of scenario 1.

Moreover, the salt concentrations at 10 MLs were recorded after the SEAWAT simulation with increased hydraulic conductivities, porosities and BWs′. Only three-time step data was recorded to minimise the computational burden. A bar graph was plotted to compare the changes in the salt concentrations at various MLs with the controlled simulated results. The comparison of different salt concentrations at different MLs is shown in Fig. 5b. MLs 1–5 and ML 10 showed the changes in the concentrations, whereas MLs 6–9 did not show any significant changes bearing the concentration values close to 0 kg/m^3^. The salt concentration decreased vastly at all MLs when the hydraulic conductivity and porosity increased.

The salt concentration at ML1 for the controlled simulated result was 32.248 kg/m^3^, whereas the simulated concentration at ML 1 for this scenario was 0.0388 kg/m^3^ for a 10-year time step. The difference in these two concentrations resulted in a 99.88% decrease in the salt concentration. Moreover, there was a 63.34% decrease at the 20-year time step and an 18.83% decrease at the 30-year time step for ML 1. Similarly, for ML 2, a 99.99%, 99.46% and 70.43% decrease in salt concentrations for 10-, 20-, and 30-year time steps was observed, respectively. For ML 3 and 4 at the 10- and 20-year time step, the concentrations decreased by more than 90%. However, the salt concentration for ML 3 at 30-year time steps decreased by 60.9%, and for ML 5, it decreased by 30.04%. A similar range of % decrease was noted for ML 5. For ML 10, at 10–20-30-year time steps, the salt concentration was decreased by 85.19%, 13.52% and 0.30%, respectively. Moreover, with increasing time, the difference in the salt concentrations at various MLs will be very less. To achieve this, we have to run our model for a longer time. However, in real life, there are a lot of uncertainties; hence, prediction becomes challenging.

### Scenario 3: Increasing the recharge rate

The initial recharge value for the entire model domain was 0.0004 m/day. However, in this scenario, we have increased the recharge rate into the model domain by 50%. The new recharge value for this scenario was 0.0008 m/day. All the other parameters and boundary conditions remained the same. We have also reduced the BWs to 5 as this was the initial set-up for the controlled or illustrative study area. The plan view of the salt concentrations at 3 different time steps for this scenario is shown in Fig. 3(a-10 to a-30). The controlled results indicate that the salt concentrations for the coastal MLs (MLs 1–5, 10) are ≈ 35 kg/m^3^, as shown in Fig. 2(a-10 to a-30). The significant changes in the SWI show after 20 years as the saltwater concentrations are lower than 30 kg/m^3^ for the coastal side MLs.

The side view of the saltwater movement into the coastal aquifer for this scenario was viewed from ML 1, and the results are shown in Fig. 4(d-10 to d-30). The side view can be easily compared with other scenarios. However, we have used the controlled side view to interpret our results. From Fig. 4(d-10 to d-30), it can be seen that the salt concentrations at various grids around the ML 1 have decreased. The brackish water zone was also increased, as shown on the left side of Fig. 4(d-10). The salt concentrations at 10 MLs were recorded for 3-time steps and compared with the initial simulated concentrations. A bar graph was also plotted for this scenario, and the results are shown in Fig. 5c.

The bar graph was only significant for MLs 1–5 and 10, whereas MLs 6–9 had salt concentration values very close to 0 kg/m^3^. From the bar graph, it can be seen that for all time steps, the concentration decreases if we increase the recharge by 50%. The 10-year time step showed a considerable decrease in salt concentrations, with MLs 4 and 5 showing 81.98% and 78.79%, respectively. The 20-year and 30-year time steps for MLs 4–5 portrayed a 16.4% and 9.3% decrease, respectively. Similarly, other MLs showed a considerable percentage decrease in salt concentration for a 10-year time step, whereas, for 20-year and 30-year, the percentage decrease was not higher than 20%.

### Scenario 4: Installation of injection wells

The number of BWs was 5 in the initial model set-up, and the same number of injection wells (IWs) was placed uniformly along the coastal boundary. IWs act as the groundwater system’s source, adding water to the aquifers and increasing the total water volume. IWs were placed in the exact location where we placed extra BWs and marked as BW′, as shown in Fig. 1. The volume of water injected into the aquifer was 12,000 m^3^/day and was constant for the 30-year stress period. The plan view of the simulated results of this scenario is shown in Fig. 3(b-10 to b-30). The results were compared with the controlled simulated results. Adding IWs shows significant changes in saltwater flow into the aquifer system. The continuous pumping using PWs and injecting using IWs did not allow the saltwater to reach PWs 13, 14, and 15, even after 30 years.

The side view of ML 1 was also captured to see the saltwater movement after adding IWs, and the results are shown in Fig. 4(e-10 to e-30). The results indicate that the salt water did not reach ML 1 for 10–20-30-year steps. Moreover, the brackish water zones were also formed, as shown on the far left side of all bars in the side view for this scenario. The IWs significantly reduced the salt concentrations around the surrounding area. It forms a barrier, and approximately within 1 km of range, the saltwater does not come close to the PWs.

The salt concentrations were also recorded at various MLs after simulating the model using SEAWAT modules. The simulated results from this scenario were compared with the initial results. The salt concentrations for 10 MLs from this scenario and the controlled salt concentrations are shown in Fig. 5d. MLs 1–5 and 10 showed the changes, whereas MLs 6–9 had salt concentrations of ≈ 0 kg/m^3^. The salt concentration for all 3-time steps at ML 1 was 0.00067 kg/m^3^. A significant 99.99% decrease in the salt concentration was noted compared with the initial simulated salt concentrations. Similar results were noted at ML 2 and ML 4. ML 3 had an 86.31% decrease for a 10-year time step, whereas the 20-year time step showed a 13.59% decrease, and the 30-year time step showed a 0.98% decrease. ML 10 showed an 8.6% decrease in salt concentrations at 10-year time steps, whereas it did not show significant variations in the salt concentrations for 20- and 30-year time steps.

### Scenario 5: Increasing the hydraulic head

The hydraulic head was raised on the coastal boundary by 5 m. The final coastal head values for the eastern and western nodes were 186 m, respectively. The other head values of other boundaries remained the same. Also, other hydrogeological parameters remained the same. Initially, the head was raised by 1 m. However, no significant change was noted in the simulated results. Hence, it was raised by a significant amount in this scenario. The plan view of the simulated results is shown in Fig. 3(c-10 to c-30). The current result from this scenario was compared with the initial simulated results. The results indicate that raising the head on the coastal boundary allows the saltwater to flow more easily into the model domain or the aquifer. All 3-time steps, 10–20-30-year, showed that saltwater had moved further into the model domain when compared with the model where the heads were not raised.

The side view of the saltwater movement was also viewed at ML 1, and the results are shown in Fig. 4(f-10 to f-30). The bars from 4(a-10 to a-30) differentiate the salt concentrations at all 3-time steps. More saltwater has moved into the model domain, as shown from the side view as well. On the seaward of ML 1 grids, the salt concentrations at all 3-time steps show a considerable salt concentration of 39.5 kg/m^3^, marked in red. Increasing the hydraulic head shows significant changes in the salt concentration compared with other scenarios, as shown in Fig. 4.

Similar to other scenarios, after changing the head values at the coastal boundary, the simulation was performed using the SEAWAT module. The new simulated salt concentrations were compared with the initial simulated salt concentrations at various locations in the study area. Salt concentrations at 10 MLs were compared using bar graphs, as shown in Fig. 5e. The salt concentrations at MLs 1–5 and 10 were significant, whereas the salt concentrations at ML 6 were slightly significant only for the 30-year time step. MLs 7–9 had salt concentration values of ≈ 0 kg/m^3^. The salt concentration at ML 1 at 10–20-30-year steps increased by 4.93%, 0.79% and 0.81%, respectively. At ML 2, the salt concentration for a 10-year tie step was ≈ 0 kg/m^3^, whereas, with time, the salt concentration at a 20-year time step increased by 63.9% compared with the initially simulated salt concentrations at these locations. However, a slight increase in the concentration was noted for the 30-year time step for ML 2.

The concentration at ML 3 for a 10-year time step was 4.01 kg/m^3^, whereas the controlled simulated concentration was 0.00053 kg/m^3^. For 20-year and 30-year time steps, the concentration increased by 11.78% and 2.19%, respectively. For ML 4 at 10–20-30-year time steps, the salt concentration increased by 607%, 4.66% and 2.18%, respectively. For ML 5 at 10–20-30-year time steps, the salt concentration increased by 701%, 5.94% and 2.47%, respectively. For ML 6, the concentration at the 30-year time step was 0.89 kg/m^3^, whereas, at other time steps, it was ≈ 0 kg/m^3^. The ML 10 showed fewer variations in the concentrations for this scenario. However, the concentrations were increased by 12.86%, 1.62%, and 1.70% for the 10–20-30-year time steps, respectively.

### Scenario 6: Changing the model grid size

**Fig. 6:**
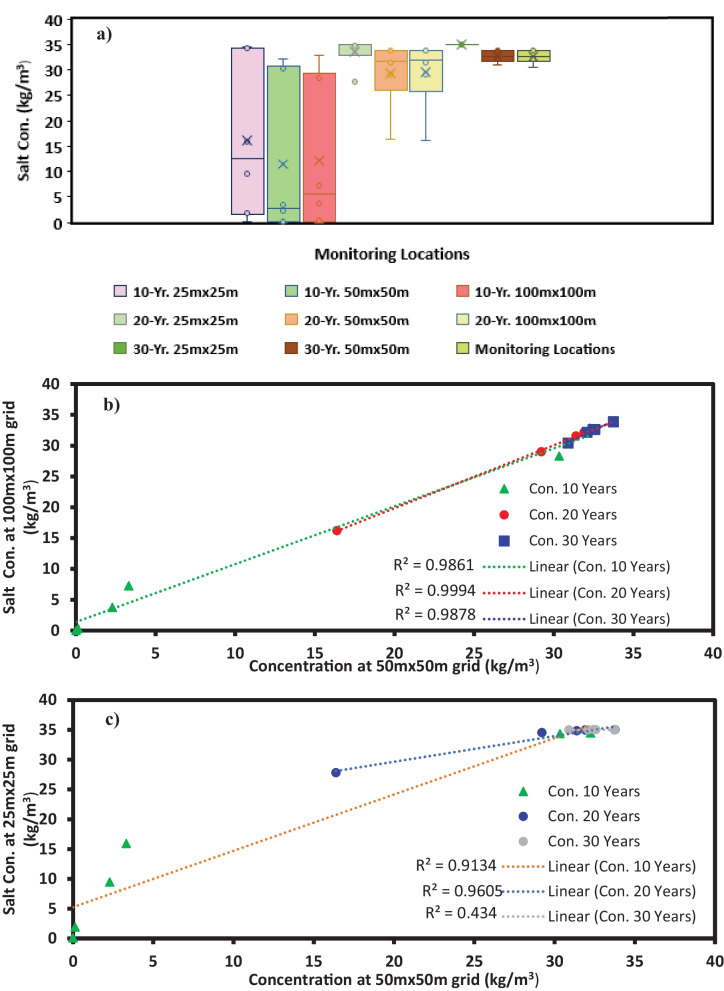
**a**–**c** shows the box plot of simulated salt concentrations obtained by using different grid sizes, the linear correlation of 100 m × 100 m and 50 m × 50 m grid results, and the linear correlation of 50 m × 50 m and 25 m × 25 m grid results, respectively

The initial grid size for developing the numerical simulation model was 50 m × 50 m. In this scenario, two different grid sizes were used to discretise the model domain. Firstly, the grid size was reduced to 25 m × 25 m, and the salt concentrations at 10 MLs were recorded. Secondly, the grid size was increased to 100 m × 100 m, and the salt concentrations were again recorded for all MLs. The different salt concentrations at two different grid sizes were compared with the 50 m × 50 m grid salt concentrations. The box-and-whisker plot was produced to compare all three grid-size sensitivities, and the results are shown in Fig. 6a.

The box-and-whisker plot for all three grid sizes at 10–20-30-year time steps for 10 MLs is plotted side by side. For the 10-year time step, the mean values for 25 m × 25 m, 50 m × 50 m and 100 m × 100 m are 16.03 kg/m^3^, 11.38 kg/m^3^ and 12.07 kg/m^3^, respectively. For the 20-year time step, the mean values for 25 m × 25 m, 50 m × 50 m and 100 m × 100 m are 33.68 kg/m^3^, 29.40 kg/m^3^ and 29.45 kg/m^3^, respectively. For the 30-year time step, the mean values for 25 m × 25 m, 50 m × 50 m and 100 m × 100 m are 34.99 kg/m^3^, 32.58 kg/m^3^ and 32.59 kg/m^3^, respectively. Moreover, one outlier was noted for the 25 m × 25 m grid at a 20-year time step. This outlier was from ML 2, with a value of 27.79 kg/m^3^. All other values were within the 34 to 35 kg/m^3^ range.

The regression correlation between the 25 m × 25 m, 50 m × 50 m and 100 m × 10 0 m grid size and the changes in the concentrations at each MLs were also analysed, and the results are shown in Fig. 6b, c. The correlation between the 50 m × 50 m and 100 m × 100 m grid shows that for 10–20-30-year time steps, the coefficient of determination (*R*^*2*^) value was 0.986, 0.999 and 0.987, respectively. Moreover, the correlation between the 50 m × 50 m and 25 m × 25 m grid shows that for 10–20-30-year time steps, *R*^*2*^ value was 0.913, 0.961 and 0.434, respectively. The low value of *R*^*2*^ was observed for the correlation between the 30-year salt concentrations of 50 m × 50 m and 25 m × 25 m. However, the average percentage change between the two sets of concentrations at ML 1–5 and 10 was 7.49%. Moreover, the time taken by the model to converge for 25 m × 25 m, 50 m × 50 m and 100 m × 100 m were 57 min, 5 min and 1 min, respectively.

### Scenario 7: Multi-species contaminant transport

The numerical simulation model was made more complex by adding two contaminants towards the northern and eastern sides of the model domain, as shown in Fig. 1. An industrial dumping site was at the northern side of the model domain, and some fluids were leaking into the ground due to the cracks in the industrial concrete dump. Similarly, there was a big automotive workshop towards the eastern side of the study area, near river 1. The automotive workshop does car servicing, and due to improper disposal of automotive engine oil, some leak into the ground. For this scenario, we used SEAWAT to simulate the leachate from the industrial dump site and oil leakage from the automotive workshop into the illustrative study area. The parameters used to solve the coupled flow and transport equations for two different species are given in Table 3.

**Fig. 7:**
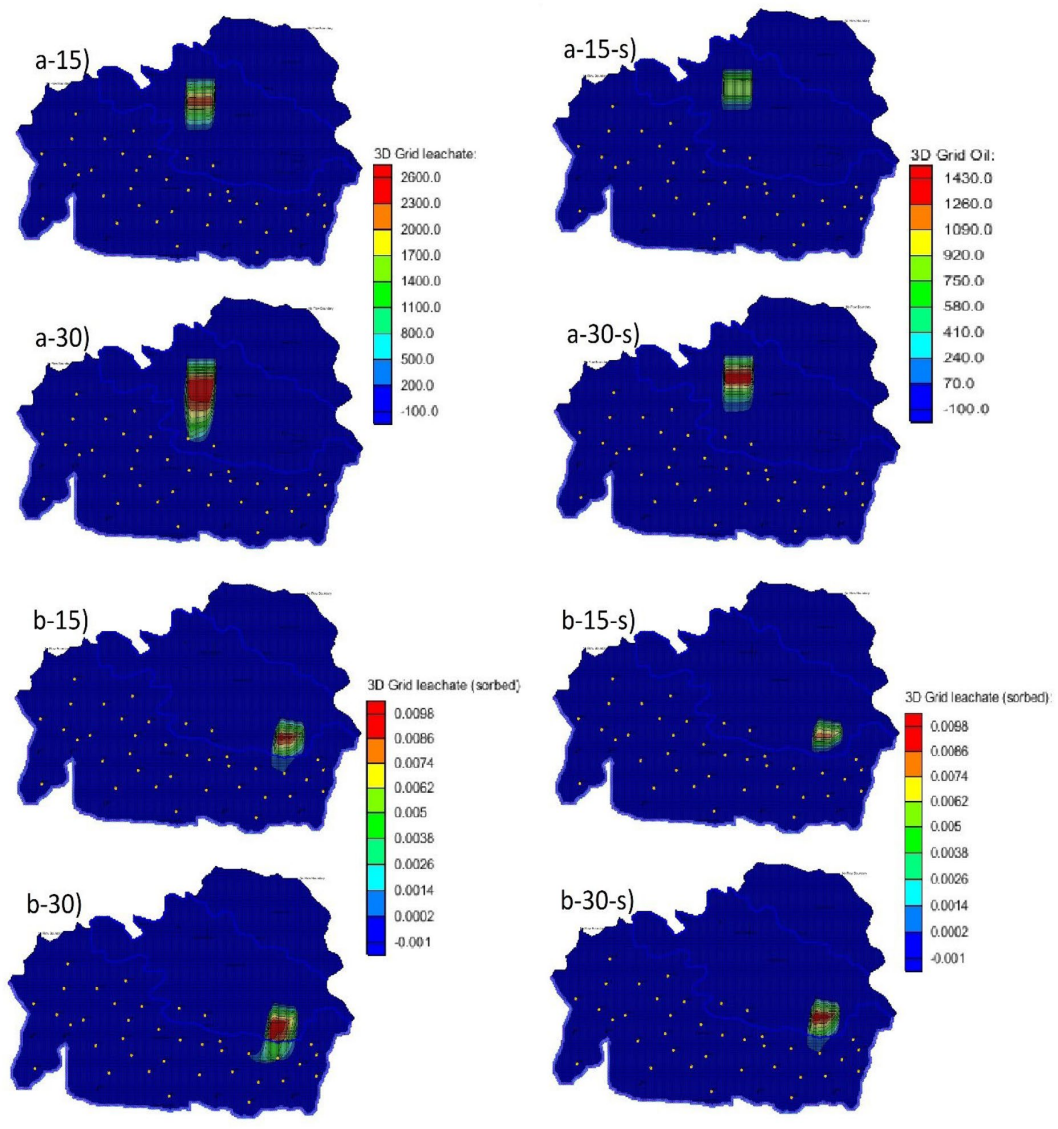
(**a**-**15**–**a**-**30**) shows the leachate concentrations for 15- and 30-year time steps; (**a**-**15** **s**–**a**-**30** **s**) shows the leachate concentrations for 15- and 30-year time steps after sorption and chemical reactions. (**b**-**15**–**b**-**30**) shows the engine oil concentrations for 15- and 30-year time steps; (**b**-**15** **s**–**b**-**30** **s**) shows the engine oil concentrations for 15- and 30-year time steps after sorption and chemical reactions

The stress period for leachate and oil contaminants was 30 years. Simultaneously solving the flow and transport equations at every node, the SEAWAT simulated the multi-species contaminants, and the results are shown in Fig. 7. Figure 7 (a-15 and a-30) are the simulated results for leachate at 15-year and 30-year time steps, respectively. As the stress period increases, the leachate spreads and moves with the groundwater flow in the unconfined aquifer. The leachate reached PW3 after 30 years and contaminated the well with a leachate concentration of ≈ 600 kg/m^3^. Similarly, Fig. 7 (b-15 and b-30) are the simulated results for engine oils at 15-year and 30-year time steps, respectively. Parts of river 1 and PW11 were slightly contaminated at a 15-year time step with engine oils. However, at a 30-year time step, the parts of river 1, PW11 and PW6 were highly contaminated with engine oils. The concentration of oil at PW6 and PW11 was 450 kg/m^3^ and 283 kg/m^3^, respectively.

Leachate and engine oil were initially simulated with advection, dispersion, sources and sink packages from MT3DMS modules. However, in reality, some solutes get sorbed and dissolved into the groundwater system. Hence, we also included chemical reaction packages in MT3DMS, and the results are shown in Figs. 7 (a-15-s to b-30-s), where -15–30 indicates the time steps and -s indicates the inclusion of sorption in the simulated results. Sorption is when the liquid contaminants get attached to the charged soil particles. The bond between the ions can be weak or covalent bonds. The sorption process makes the contaminants travel and disperse at much slower rates. Figure 7 (a-15-s and a-30-s) demonstrate sorption. Including the sorption process, the water from PW3 is safe even after 30-year time steps. Similarly, Fig. 7 (b-15-s and b-30-s) demonstrate the sorbed oil in the aquifer. After 30 years, the PW6 was not affected. However, the engine oil has reached near PW11, and after a few more years, it will contaminate the well.

## Discussion

The SWI in the coastal aquifer is a significant concern for PICs. To manage and control the SWI, an illustrative study area in the context of PICs was considered for this study. Different scenarios were tested that affect the SWI in coastal aquifers. The scenarios presented in this study were the first for PICs. Moreover, the multi-species contaminants were modelled to see how they affect the freshwater wells. Based on these scenarios, the governing bodies can draft and implement the SWI and other contaminant management policies.

The scenarios that effectively showed the decline in SWI in coastal aquifers were increasing the injection wells (IWs) and the aquifer’s recharge. The salt concentration and SWI are highly sensitive to hydraulic conductivity as well. The increase in barrier wells (BWs) was also helpful in controlling the SWI. Armanuos et al. (2020) studied how the BWs control SWI in sloping unconfined aquifers and found that BWs are very effective in facilitating the retreat of SWI in coastal aquifers. Similarly, our results also indicate that SWIs are controlled by adding BWs. However, the placement of barrier wells has to be carefully considered. In the current study, we uniformly placed the BWs in the model domain, which reduced the SWI in some areas, whereas some areas had increased SWIs.

The BWs simulated results in plan view showed that the hydraulic gradient is towards the sea. Hence, the saltwater moves away from the BWs and flows in a different direction, eventually causing the concentrations at MLs to increase. However, in other locations, the salt concentrations have decreased. Therefore, introducing BWs and the placement of BWs into the model domain is essential. Hence, we can simulate the best possible locations for placing and digging BWs to prevent the SWI and contaminating the freshwater wells using trial-and-error methods. Optimization methods can also be used to find the optimal locations of BWs that can manage the SWIs; however, we have not included this component in this limited study. Therefore, the placement of barrier wells is essential if we want to use BWs to control SWI in coastal aquifers. However, BWs sometimes remove more freshwater than salty water, decreasing freshwater resources (Pool & Carrera, 2010). Also, the disposal of abstracted saline water can also become an issue (Kumar, 2006).

Adding the injection wells (IWs) can effectively control the SWI if placed in the correct locations. IWs can reduce the SWI and change the saline water flow directions. However, we can place the injection wells near the toe once we know the toe of the saltwater wedge through modelling. Luyun and Momii (2011) reported that IWs would be more effective if placed at the saltwater wedge’s toe. The authors also reported that deeper barrier penetration and closer to the coast would effectively control SWI. Similarly, in our study, our modelled results showed that the BWs and IWs placed closer to the coast and near the toe of the saltwater wedge effectively reduced salt concentrations in various monitoring location wells (MLs). However, the addition of BWs and IWs can be costly and time-consuming.

The recharge is also a vital boundary condition that needs to be considered when modelling and pumping groundwater. Sometimes the recharge can be the only source to the system that can keep the freshwater volume within limits and prevent the wells from drying up. In one of the scenarios, we increased the recharge of the entire model domain by 50%, and the results were quite fascinating. The salt concentration at various MLs was reduced. Hence increasing the recharge reduces the SWI. Similar results were also reported by Motallebian et al. (2019) and Hussain et al. (2019).

The model sensitivity of hydraulic conductivity and porosities was also tested, where it was found that the higher the hydraulic conductivity, the lesser the saltwater moves into the model domain. We also reduced the hydraulic conductivities and simulated the results. It showed that the SWI encroachment into the model domain was increased. The increase was shown because the hydraulic gradient was from the northern side of the model to the southern side (coastal boundary). The head was lower in the coastal boundary. Hence, the saltwater travelled less if we increased the hydraulic conductivity. Also, the higher amounts of pumping enabled a lot of drawdowns, which must have reduced the SWI rates. However, once we changed the head of the coastal side to be higher than the northern boundary, the results were the opposite.

In our study, we found that if the hydraulic gradient is from land-ocean and has significant pumping wells, increasing the hydraulic conductivity will decrease the SWI. If the hydraulic gradient is from ocean-land and has significant pumping wells, increasing the hydraulic conductivity will increase the SWI. The increased hydraulic conductivities resulted in higher SWI, and decreased hydraulic conductivities reduced SWI. The reduction in SWI by reducing the hydraulic conductivities was also reported by Dror et al. (2004) and Strack et al. (2016). Both these studies used laboratory experiment set-ups. Hence, in our study, we have used numerical models to demonstrate similar effects.

Moreover, the hydraulic gradient plays a vital role in SWI encroachments, as shown in our results. During the initial model design, modellers must carefully incorporate the hydraulic head values based on the field observations. This will help to provide better arguments on whether to increase or decrease the hydraulic conductivities to control the SWI. Hydraulic conductivities can be artificially increased or decreased by injecting the compressed air into the soil consisting of the coastal aquifers (Dror et al., 2004). Christiansen (1944) earlier reported that the trapped air can significantly reduce the hydraulic conductivities, whereas the loss of trapped air can significantly increase the hydraulic conductivities of soil. Changing the hydraulic conductivities will have significant effects on SWI in coastal aquifers.

Climate change and sea-level rise (SLR) were also considered in this study, where the hydraulic heads of the coastal side were increased due to increasing sea levels. The global projected SLR is ≈ 0.3 m by the year 2050 (Rebecca, 2022). Initially, we increased our heads by adding 0.3 m to the coastal heads, then increasing it by 1 m. The results did not show significant variations in the salt concentrations at various MLs if the heads were increased to 1 m. Loaiciga et al. (2011) also tested the SLR scenarios within 0.5–1 m and reported that SLR would have minor contributions to SWIs. However, to see the actual SWIs due to SLR, we increased the heads by 5 m, and then the SWI was significant. Increasing the heads on the coastal boundaries allows more SWI encroachments into the model domain. Therefore, an increase in sea level by a significant amount only contributes to SWI. However, at the current global SLR projections, the SWIs are not significant. Hence, climate change and SLR may increase the SWIs for coastal aquifers in the future.

The sensitivity of grid size to the final model simulated results was also tested. Changes in grid size (discretisation) do influence numerical simulation results. Therefore, it is essential to ensure that discretisation does not vary the simulation results by more than an acceptable (e.g., 5–10%) range. The current results revealed that if we increase or decrease the grid size, the simulated results did not vary more than 10%. However, the computational burden was increased when the grid size was decreased. It took approximately 57 min for the model to converge. The grids have to be selected based on the accuracy of model convergence, computational time, and outputs should not vary much. Moreover, according to Barnett et al. (2012), if the grids are refined locally, the increase in node spacing or grid size between adjacent elements must be maintained in limits to avoid computational burdens with significant contrasts in cell size. The authors also stated that as a rule of thumb, a factor of 1.5 should be kept as the maximum ratio of the volumes of neighbouring cells. Hence, for the current study, 50 m × 50 m cells were ideal in computations.

Studies on multi-species contaminant using SEAWAT in PICs have not been done so far. Also, to the best of our knowledge, very few studies have been conducted showing SWI and other contaminants. However, this is the first study where SWI and the other two species are modelled using SEAWAT. In the current study, we used salt, leachate and engine oils as contaminants and modelled them using dispersion, advection and chemical reaction packages inbuilt the latest version of SEAWAT. We were trying to model the three species affecting the freshwater lens with time. The saltwater intrusion, leachate and engine oil plumes were clearly shown in the results section. However, as shown in our results, sorption and adsorption rates were also added to the leachate and engine oils, which reduced the plume size. Al-Hashimi et al. (2021) reported that sorption, adsorption and other chemical reactions reduce the contaminations and can help in remediations.

## Conclusion

The groundwater modelling for an illustrative study area in the context of Pacific Island countries (PICs) is presented in this paper. Actual aquifer and pumping locations were treated as the illustrative study area because the hydraulic head and enough pumping data were unavailable to calibrate and validate the model. However, some of the hydrogeologic parameters and boundary conditions were realistic. The multi-species solute and heat transport module SEAWAT was used to model the study area. Seven different scenarios were tested using SEAWAT. The scenarios presented here and the different sensitivity analysis conducted in this study have never been conducted for PICs. The model and the simulated results were sensitive to various parameters presented in this study. The main aim of the scenario testing was to manage or control the saltwater intrusion into the current coastal aquifer.

The scenarios that provided the best saltwater intrusion management strategies were using injection wells to pump freshwater near the toe of the saltwater wedge, increasing the recharge of the system, using barrier wells and decreasing or increasing the hydraulic conductivities. The hydraulic gradient will determine whether to increase or decrease the hydraulic conductivities to manage SWI. The location of placing the barrier wells is essential, and it can be achieved by placing the wells within the model domain using the trial-and-error method and simulating the lowest salt concentrations at various monitoring locations and pumping wells. Furthermore, the sea-level rise by 0.3 to 1 m did not substantially impact SWI encroachments. Moreover, it is essential to ensure that changes in the grid size do not vary the simulation results by more than an acceptable (5–10%) range.

Multi-species contaminants can be modelled using SEAWAT, and different packages can be used to see the flow, direction and size of the contaminant plumes. We established that the application of advection, sorption, adsorption and other chemical reactions slows the flow velocity and direction of the contaminant plumes. The limitations of this study were that we did not have field hydraulic head, pumping and water concentrations at various locations. We could have calibrated and validated our model if we had these data. However, PICs do not have all the required data to develop a numerical model using SEAWAT. Moreover, PICs do not have data for other contaminants like engine oil and leachate. PICs need funding and expertise for ground-level work in setting up the groundwater monitoring equipment. Hence, with the field data, we can provide better saltwater intrusion and other contaminant management strategies for PICs.

## Data Availability

The datasets used and/or analysed during the current study are available from the corresponding author on reasonable request.
